# Mindful Self-Care and Compassion Fatigue in Nurses: The Chain Mediating Roles of Resilience and Professional Identity

**DOI:** 10.1155/jonm/8572654

**Published:** 2025-02-11

**Authors:** Junfan Wei, Zhengcheng Yun, Yang Zhang, Yongqi Liang, Ziping Hu, Chanchan Gao, Xiaona Tang, Hanjiao Liu

**Affiliations:** ^1^The Seventh Clinical Medical College, Guangzhou University of Chinese Medicine, Shenzhen, China; ^2^School of Medicine, Southeast University, Nanjing, China; ^3^Department of Oncology, Zhongda Hospital Affiliated to Southeast University, Nanjing, China; ^4^Department of Nursing, Shenzhen Hospital of Integrated Traditional Chinese and Western Medicine, Shenzhen, China

**Keywords:** compassion fatigue, mindful self-care, professional identity, resilience

## Abstract

**Aim:** To investigate the relationship between mindful self-care and compassion fatigue and the chain mediating effects of resilience and professional identity.

**Background:** Compassion is a critical quality for nurses, but they often face the risk of compassion fatigue.

**Methods:** From October 2023 to May 2024, a cross-sectional survey was conducted in six tertiary hospitals in Guangdong, Henan, and Jiangsu provinces by convenience sampling. A total of 1315 clinical nurses in the hospital were surveyed using the Social–demographic Characteristics Questionnaire, the Chinese Version of the Brief Mindful Self-Care Scale, the Chinese Version of the Connor–Davidson Resilience Scale, the Professional Identity Scale for Nurses, and the Chinese Version of the Compassion Fatigue Short Scale. The mediation model was analyzed using a bias-corrected bootstrapping method with PROCESS 4.1 implemented in SPSS 26.0.

**Results:** Pearson correlation analysis showed that there was a positive correlation between nurses' mindful self-care, resilience, and professional identity. Mindful self-care, resilience, and professional identity were negatively correlated with compassion fatigue. Resilience played a partial mediating role between mindful self-care and compassion fatigue. Professional identity also played a partial mediating role between mindful self-care and compassion fatigue; mindful self-care affected compassion fatigue through resilience and professional identity.

**Conclusion:** Nurses' mindful self-care can affect compassion fatigue through the mediating role of resilience and professional identity. In the future, attention should be paid to cultivating nurses' mindful self-care ability and resilience and improving nurses' professional identity, which may help to reduce nurses' compassion fatigue.

**Implications:** Hospital leaders should actively pay attention to the mental health of nurses and take measures to improve their ability of mindful self-care, which may help to prevent compassion fatigue and improve nursing quality.

**Reporting Method:** The study adheres to the STROBE reporting guidelines.

**Patient or Public Contribution:** No patient or public contribution.

## 1. Introduction

Compassion is the primary ethical principle for nursing staff to provide quality care [1]. It is also an essential moral quality for nurses to possess compassion [2] and is recognized as the “most precious asset of nursing” [3]. The International Council of Nursing (ICN) emphasizes the importance of compassion by incorporating it as one of the five core values of nursing (“International Council of Nursing [4]. The ICN code of ethics for nurses. Retrieved from https://www.icn.ch/who-we-are/code-of-ethics-for-nurses/,”). Studies have found that nurses with more compassion are more likely to gain the trust of patients [5], help patients recover faster, and improve their own job satisfaction [6]. So it is important for nurses to be compassionate, which has become a common societal expectation for nurses [7]. Despite that the critical role compassion plays in ensuring quality care and personal development of nurses, compassion fatigue among nurses is widespread. According to the results of a survey, about 2/5 clinical nurses suffer from compassion fatigue at work [8]. Therefore, understanding the factors influencing compassion fatigue in nurses is critical to help nursing administrators develop effective measures to prevent and alleviate compassion fatigue in nurses.

Compassion fatigue refers to a psychological and behavioral phenomenon where caregivers experience reduced interest and ability to empathize due to prolonged exposure to the suffering of others and is therefore also referred to as “the cost of care” [9]. A person experiencing compassion fatigue often exhibits fatigue or decreased energy and a lack of compassionate capacity [3]. Compassion fatigue in the field of nursing refers to a series of negative physical, emotional, spiritual, and social reactions that ultimately arise as a result of a nurse's repeated exposure to patient's suffering, high-stress environments, constant self-devotion, long-term neglect of accumulated stresses, and his or her own emotional demands, which leads to compassion stress that exceeds the nurse's level of endurance [10]. Compassion fatigue among nurses significantly reduces patient safety and quality of care, negatively affects nurses' mental health and life satisfaction [11], and is associated with higher turnover intentions [12]. For nurses, compassion fatigue is considered an occupational hazard difficult to avoid for several reasons [10]. Firstly, as direct providers of healthcare services, nursing staff have almost the most frequent contact with patients, and the characteristics of their profession make it difficult for them to avoid self-devotion and exposure to patients' suffering [13]. Secondly, nurses are always under high-pressure environment due to labor shortages [14], shift work, and patients' emergencies. In this situation where other factors contributing to the impact of compassion fatigue are difficult to be addressed, it seems crucial for nurses to actively aware their accumulated stress and emotional demands and take actions to meet their own needs to prevent and alleviate compassion fatigue [9, 10]. The concept of mindful self-care seems to provide new insights into overcoming these challenges, which may present a promising approach to alleviating compassion fatigue. Previous studies in several different populations have shown that mindful self-care has the potential to reduce the risk of professional burnout [15, 16]. However, as a special form of nurses' professional burnout [17], it is still unclear whether nurses' compassion fatigue will also be affected by mindful self-care and its underlying psychological mechanism. These research gaps in the literature emphasize the need for targeted studies exploring the role of mindful self-care in alleviating compassion fatigue in nurses. Addressing these gaps is critical for in-depth exploration of the relationship between mindful self-care and compassion fatigue and potentially provides insights for further improvement of the theoretical framework and the development of interventions to effectively alleviate compassion fatigue. Therefore, this study focused on the association between mindful self-care and compassion fatigue in nurses and explored the potential mediation mechanism underlying this association.

### 1.1. Mindful Self-Care and Compassion Fatigue

The concept of mindful self-care was developed by Cook-Cottone by incorporating elements of mindfulness into traditional self-care, which emphasizes the importance of enhancing self-awareness through mindfulness and subsequently leads to intentionally engaging in activities that meet one's own needs in order to promote holistic well-being [18]. Mindful self-care is a practice process that integrates personal internal and external needs based on mindful thinking to conduct conscious self-care to improve personal well-being [19]. It is described as a continuous and dynamic process of practice, with the first step being the active identification and assessment of an individual's internal and external needs and the second step being the intentional engagement in self-care practices that serve the individual's needs in order to support the individual's well-being [19]. Before the influence of mindfulness on self-care, traditional models of self-care, while useful, sometimes regarded self-care as a task and even brought additional stress [20]. Moreover, previous research in the field of self-care has the problem that although the importance of self-care for nurses is emphasized, there is a lack of practical guidance [21]. For example, nurses, who are often busy in their daily work and life, are accustomed to prioritizing the needs of their patients for a long time and lack attention to themselves, thus making it difficult for them to identify their true needs despite being aware of the importance of self-care. The incorporation of the concept of mindfulness can help individuals become more aware of their needs in order for nurses to make more conscious choices and adjustments to their self-care activities [22]. Therefore, mindful self-care can be considered an improved or deepened form of self-care. These two concepts are essentially similar and have the same core goal of maintaining and enhancing the physical and mental health and well-being of individuals. The two concepts are distinguished by certain differences in methodology, practice, and focus.

The mindful self-care and helping model [16] suggests that mindful self-care practices have function of addressing fatigue within the occupational setting, especially among helping professionals. It is thought that people who practice more mindful self-care are more likely to be aware of and address their unsatisfied needs and reduce burnout and secondary traumatic stress, which may lead to compassion fatigue [17, 19]. Numerous empirical studies have shown that the potential mitigation effect of mindful self-care on compassion fatigue covers the strongest protective factor against burnout and secondary traumatic stress among hospice professionals [23] and chaplains [24]. Although there were clues in these studies that could infer a correlation between mindful self-care and compassion fatigue, the impact of mindful self-care on compassion fatigue in nurses has not been robustly determined. Based on the above theoretical and empirical considerations, we propose the following hypothesis.

**Hypothesis 1 .**

Mindful self-care is negatively associated with compassion fatigue in nurses.

### 1.2. Mindful Self-Care, Resilience, and Compassion Fatigue

Understanding how mindful self-care affects compassion fatigue is important for nursing managers developing effective strategies to reduce nurses' compassion fatigue. Resilience refers to a person's ability to recover from adversity in the face of negative life events such as trauma and disaster [25], and this ability is essential to protect a person's mental health and reduce the negative impact of stress [26]. According to the job demands–resources (JD-R) model [27], when employees face excessive job demands, adequate resources can alleviate the negative impact of high demands on employees' physical and mental health. As research has progressed, psychological capital (PsyCap), such as resilience, has been incorporated into work resources and shown to be protective against burnout [28]. When individuals are more resilient, they are more likely to recover from the negative effects of compassion stress and experience less compassion fatigue [29]. Empirical research has consistently shown that resilience plays a crucial role in protecting nurses from compassion fatigue [30, 31].

Resilience as a valuable trait can be cultivated through the practice of mindful self-care, which helps individuals remain aware and acceptable in the face of stress or negative emotions, thereby reducing the accumulation of negative emotions and improving emotional regulation [19]. Previous studies have proved that there are positive correlations between mindful self-care and resilience among palliative care providers [32] and nurses [33]. At the same time, mindful self-care may help individuals build more positive experiences and a sense of value at work, such as better performance through positive emotion regulation [18]. These positive outcomes may further strengthen the individual's psychological resource reserves, including resilience, enabling them to better resist compassion fatigue.

Therefore, based on these theoretical insights and empirical findings, we propose the following hypothesis.

**Hypothesis 2 .**

Resilience mediates the relationship between mindful self-care and compassion fatigue.

### 1.3. Mindful Self-Care, Professional Identity, and Compassion Fatigue

Professional identity is an individual's cognition and opinion on the influence, importance, and belief of own profession based on skills, knowledge, values, moral concepts, personal identity, group identity, and social background [34]. In a professional environment that demands a high degree of compassion, the professional identity may be effective in alleviating emotional exhaustion and reducing the occurrence of compassion fatigue by increasing the sense of professional worth and belonging. Social Identity Theory [35] suggests that professional identity helps individuals gain a sense of meaning and satisfaction from their work, thus counteracting some of the negative effects of occupational stress (e.g., compassion fatigue due to the long-term demands of compassion in nurses' work). A study of nursing interns further revealed that professional identity partially mediated the effect of moral distress on compassion fatigue and that increased professional identity contributed to reduced compassion fatigue [36]. The reason for this protective effect is that compassion fatigue often stems from prolonged exposure to compassion-demanding work environments, whereas a well-established professional identity provides practitioners with a stronger sense of purpose and belonging, thus reducing the possibility of adverse effects [37].

On the other hand, it has been proposed that mindful self-care improves an individual's ability to be aware of emotions and the environment and enhances self-regulation [16], which may help individuals perceive their occupational roles in a positive light and enable individuals to cope with occupational challenges more effectively, thus to improve the quality of occupational life, which has also been shown to be positively associated with professional identity [38]. Empirical research findings also reveal that mindfulness-based self-care can enhance the professional identity of healthcare professionals [39, 40]. It appears that seeming professional identity may play a role in the relationship between mindful self-care and compassion fatigue.

Therefore, based on these theoretical insights and empirical findings, we propose the following hypothesis.

**Hypothesis 3 .**

Professional identity mediates the relationship between mindful self-care and compassion fatigue.

### 1.4. Resilience and Professional Identity

With respect to the relationship between resilience and professional identity, it has been argued that resilience is a critical psychological resource that enables individuals to navigate workplace challenges, maintain emotional stability, and foster a sense of purpose, all of which contribute to the development of professional identity [41]. According to the self-consistency theory [42], professional identity reflects how individuals perceive and integrate their professional roles with their self-concept, enabling them to derive meaning and satisfaction from their work. Studies in the nursing profession have identified a positive correlation between resilience and professional identity, showing that resilience helps nurses adapt to occupational stressors and align their values with professional demands [43, 44]. For instance, resilience has been shown to buffer the negative impacts of workplace stress and improve nurses' capacity to maintain their sense of belonging and significance within their profession [44].

A recent study has demonstrated that resilience mediates the relationship between workplace adversity and professional identity by enabling individuals to recover from moral distress and workplace conflicts [45]. Similarly, resilience promotes emotional regulation and adaptive coping strategies, fostering a stronger professional identity by allowing individuals to focus on the intrinsic value of their roles [44]. Interventions designed to enhance resilience have been shown to significantly strengthen professional identity, particularly in high-stress environments such as healthcare [43]. These findings collectively underscore the critical role resilience plays in building and sustaining nurses' professional identity.

Additionally, resilience and professional identity were found to partially mediate the relationship between moral dilemmas and empathy fatigue among nursing interns [36]. Professional identity can be enhanced by increasing moral resilience, which in turn reduces compassion fatigue.

**Figure 1:**
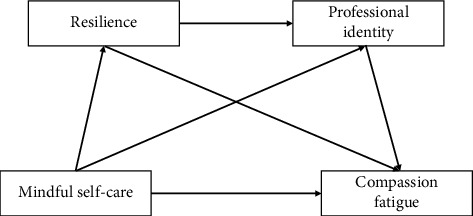
Theoretical framework of the relationship between mindful self-care and compassion fatigue, with resilience and professional identity as mediators.

Our theoretical framework, as illustrated in Figure 1, hypothesizes that mindful self-care positively influences resilience, which in turn enhances professional identity, ultimately leading to lower levels of compassion fatigue.

Therefore, based on these theoretical insights and empirical findings, we propose the following hypothesis.

**Hypothesis 4 .**

Resilience and professional identity serially mediate the link between mindful self-care and compassion fatigue.

## 2. Methods and Materials

### 2.1. Study Design

The present study was implemented using the method of convenience sampling and cross-sectional design [46], which has been widely used to investigate the status of psychological indicators in nurses. We adhered to Strengthening the Reporting of Observational Studies in Epidemiology (STROBE) guidelines and methodology in reports of cross-sectional studies (details in Supporting Table S1).

### 2.2. Sample Size

Given the observational cross-sectional study design, the following formula should be used to estimate the sample size of this study [47]:

(1) $$\begin{array}{c} n={\left(\frac{U_{\alpha /2}\sigma }{\delta }\right)}^{2}={\left(\frac{1.96\times 31.4}{3}\right)}^{2}\approx 421, \end{array}$$

where *n* represents the sample size required to estimate, *U*_*α*/2_ is the standardized normal deviation corresponding to *α* = 0.05 and 95% confidence level (*U*_*α*/2_ = 1.96, for two-tailed), *σ* represents the expected value of standard deviation in the population (*σ* = 31.4 from the pilot study), and *δ* represents the acceptable margin of error for the mean (*δ* = 3 from the pilot study). Considering the 20% invalid questionnaires, the sample size for this study should be at least 527 [48].

Participants were recruited with the help of head nurses from these hospitals. Inclusion criteria were as follows: (1) holding professional qualification certificates for nurses issued by the People's Republic of China; (2) nurses who were working in the hospital during the survey period; (3) nurses who were employed at the current hospital for more than 3 months; and (4) participating in this study voluntarily. The exclusion criteria were as follows: (1) nurses who were on vacation at the time of the survey, out for training, sick leave, or business leave; (2) nurses who could not complete the questionnaire independently; and (3) nurses who had been previously diagnosed with mental illness or drug or alcohol dependence.

### 2.3. Pilot Study

In order to ensure that the design of the questionnaire is reasonable, the questionnaire has good reliability, and to ensure that the calculation of the sample size is accurate, we carried out a pilot study. A pilot study was conducted in the first institution where the study was conducted because test–retest reliability testing was required and subjects were less likely to drop off in the first institution. According to Perneger et al. [49], the sample size of the pretest should be greater than 30. Through recruitment, a total of 51 nurses volunteered to participate in this pretest. The same questionnaire was administered to 51 nurses two weeks after the initial test for retesting [50]. The results showed that Cronbach's *α* coefficients of scales in this questionnaire were greater than 0.8, indicating good internal consistency of these scales when applied to the subjects of this study. The Pearson correlation coefficient between the first test and the retest scores of each scale was analyzed, and the results were all greater than 0.8, indicating that test–retest reliability of each scale in the questionnaire was acceptable when applied to the subjects in this study.

### 2.4. Data Collection

The convenience sampling method was used in this study. The study was conducted from October 2023 to March 2024 in six tertiary hospitals in Guangdong Province, Henan Province, and Jiangsu Province of China. Before the survey began, the research team members discussed and reached a consensus on standardized interpretation procedures, and questions may be proposed by respondents to avoid misunderstanding caused by improper explanation during the research process. In addition, all the investigators were trained in the use of the online survey tool.

Then, the research team members contacted the head nurses of some tertiary hospitals online and introduced the purpose of the study. The head nurses of six hospitals were interested in the study and were willing to assist us in the study. Subsequently, team members recruited and organized research participants with the help of the head nurse. Before conducting the survey, research team members introduced participants to the purpose of this study, stating that the questionnaire content of this study does not involve the privacy of respondents, guarantees anonymity, and indicates that participants are free to withdraw for any reason at any time.

**Figure 2:**
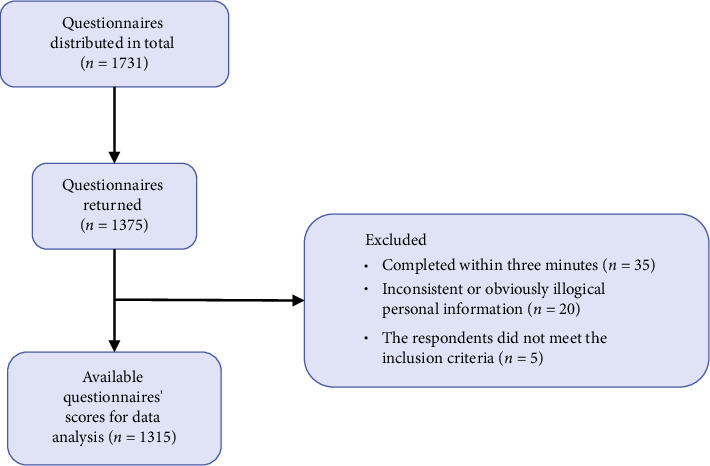
Flow chart of questionnaire result screening.

A standardized protocol was followed during the distribution of all the questionnaires. The link to the online questionnaire, which had been entered into the Wenjuanxing system (https://www.wjx.cn) in advance and had been pretested, was sent face to face to the respondents who volunteered to participate in the study. Respondents accessed the questionnaire via the link and completed it. Respondents were asked not to allow the link to the questionnaire to be sent to anyone else without permission. The contents of the questionnaire included the following: (1) Social–Demographic Characteristics Questionnaire; (2) the Chinese Version of the Brief Mindful Self-Care (BMSC) Scale; (3) the Professional Identity Scale for Nurses (PISN); (4) the Chinese Version of the Connor–Davidson Resilience Scale (C-CD-RISC); and (5) the Chinese Version of the Compassion Fatigue Scale. In order to ensure the quality of the returned questionnaires, the questionnaires were set as follows: (1) At the beginning of the questionnaire, the purpose and precautions were briefly introduced. The questionnaire had an informed consent form on the front page and an electrical signature box at the end. The respondents could draw any graph inside the box with the mouse through PC terminal access or with the finger through mobile phone access. In this survey, respondents were asked to sign inside the box. (2) In order to avoid the respondents not meeting the inclusion and exclusion criteria, the questions related to the inclusion and exclusion criteria of the study subjects were asked on the front page of the questionnaire (e.g., whether the respondents held a nursing professional qualification certificate). (3) In order to avoid incomplete returned questionnaires, all questions were set as required questions. If there are missing answers, the questionnaire cannot be successfully submitted, and the missing questions will be marked with an asterisk to remind the respondent to complete. Only the participants who met the inclusion and exclusion criteria could continue to complete the questionnaire. (4) Each IP address was allowed to fill out and submit the questionnaire only once. Ethical approval for the study was obtained from the institutional review board. Participants were assured of the confidentiality and anonymity of their responses, and data were securely stored and accessible only to the research team. In this study, we distributed 1731 questionnaires, of which 1375 were returned. Among the returned questionnaires, 35 were completed within three minutes, which is almost impossible to achieve based on the length and the time participants completed the questionnaire in the presurvey; there were 20 questionnaires with inconsistent or obviously illogical personal information; the respondents of 5 questionnaires did not meet the inclusion criteria. In order to ensure the accuracy and effectiveness, the above questionnaires were removed. Finally, 1315 valid questionnaires were collected. The effective recovery rate was 75.97%. The flow chart of questionnaire result screening is shown in Figure 2.

### 2.5. Measures

#### 2.5.1. Social–Demographic Characteristics Questionnaire

The questionnaire was designed by the researcher, and the data collected were objective, including gender, age, marital status, working years, educational attainment, organizational affiliation, professional title, position title, and night shift. The selection of these variables was based on previous theoretical and empirical studies, and we included these factors as covariates to control for possible confounding effects on the main study variables [51–53].

#### 2.5.2. The Chinese Version of the BMSC Scale

This scale is a simplified version of the mindful self-care scale and is used to measure the level of mindful self-care. The English version of the BMSC Scale was developed and validated by Cook-Cottone and Guyker [54]. The Chinese version of the scale was translated into Chinese by Yang et al. [55]. The scale contained six subscales: mindful relaxation, physical care, self-compassion and goals, supportive relationships, supportive structure, and awareness of mindfulness. These six dimensions were composed of 24 items, and each item was scored using a 5-point Likert scale. A scale of 1–5 corresponds to never, rarely, sometimes, often, and regularly. Scores range from 24 to 120. The higher the total score, the higher the level of mindful self-care. The Chinese scale has acceptable reliability and validity, and Cronbach's *α* of each dimension ranged from 0.850 to 0.933 [55]. In the current study, Cronbach's *α* of this scale was 0.942, indicating satisfactory internal reliability.

#### 2.5.3. The Chinese Version of C-CD -RISC Scale

The scale was developed by Connor and Davidson [25] to measure the resilience of respondents. According to many previous studies, the scale has been widely used in college students [56], patient caregivers [57], nurses [58], and many other groups. The Chinese version was translated and revised by Yu et al. [59] and demonstrated acceptable reliability and validity. The scale included 3 dimensions (hardiness, strength, and optimism), a total of 25 items. All items were scored on a five-point Likert scale ranging from 0 (*almost never*) to 4 (*always*). The final score of the scale is the sum of the item scores, with a higher total score indicating a higher level of resilience among the participants. In the current study, Cronbach's *α* of this scale was 0.958, indicating satisfactory internal reliability.

#### 2.5.4. The PISN Scale

The scale was developed by Liu, Zhang, and Liu [60] to measure the status of nurses' professional identity. The scale consists of five dimensions: professional self-reflection, coping style, social skills, social support, and cognitive evaluation. These 5 dimensions together formed 30 items to measure nurses' professional identity.

Each item was scored using a five-point Likert scale, with 1 point corresponding to completely inconsistent, 2 points corresponding to advantages inconsistent, 3 points corresponding to general, 4 points corresponding to relatively consistent, and 5 points corresponding to completely consistent. The sum scores of the 30 items were the total score of the scale. The higher the total scores, the higher the professional identity levels of the participants. The scale demonstrated satisfactory reliability and validity. Cronbach's *α* of each dimension ranged from 0.720 to 0.911, and Cronbach's *α* of the total scale was 0.938. In the current study, Cronbach's *α* of this scale was 0.981, indicating satisfactory internal reliability.

#### 2.5.5. The Chinese Version of the Compassion Fatigue Short Scale (C-CF-Short Scale)

The scale is simple and can effectively measure the state of compassion fatigue. The English version of the scale was developed by Adams, Figley, and Boscarino [61] by simplifying the Compassion Fatigue Scale. The Chinese version of the scale was introduced and translated into Chinese by Lou [62]. It consists of two dimensions of secondary trauma and job fatigue, including 5 items and 8 items, respectively, with a total of 13 items, using a 10-point Likert scoring method ranging from 1 (*never*) to 10 (*very frequent*). Adding the scores of each item was the total score of the scale. The total score of the scale ranged from 13 to 130, and the higher the score, the more serious the degree of compassion fatigue perceived by the subjects. Lou [62] used the Chinese version scale to conduct a survey among medical staff, and the final Cronbach's *α* coefficient of the total scale was 0.87 to 0.90. In the current study, Cronbach's *α* of this scale was 0.926, indicating satisfactory internal reliability.

### 2.6. Statistical Analysis

SPSS 24.0 (SPSS Inc., Chicago, IL, USA), a professional statistical data analysis software, was used for data analysis. First of all, descriptive statistical analysis was used to describe the social–demographic characteristics of nurses. Counting data were described using frequencies and percentages [63]. Continuous data such as the compassion fatigue score, mindful self-care score, and professional identity score were described as ($\bar{x}$ ± *s*). Secondly, Harman's single-factor tests were conducted to examine the effect of common method bias, considering that measurement was based on self-rating scales [64, 65]. Thirdly, Pearson correlation analyses were performed to determine the bivariate correlations among mindful self-care, professional identity, resilience, and compassion fatigue. Finally, the mediation model was analyzed using a bias-corrected bootstrapping method with PROCESS 4.1 implemented in SPSS [66]. To test the mediating effects of resilience and professional identity in the relationship between nurses' mindful self-care and compassion fatigue, Mode 6 was chosen for mediation analysis. We tested our hypothesized chain mediation model in which mindful self-care (X) increases resilience (M1), which affects professional identity (M2), which in turn leads to lower compassion fatigue (Y). A bias-corrected 95% confidence interval (CI) was calculated using 5000 bootstrapped resamples. A 95% CI excluding zero indicated a statistically significant mediating effect [67]. In addition, the model was controlled for covariates (gender, age, marital status, working years, educational attainment, organizational affiliation, professional title, position title, and night shift), and the study variables were standardized.

### 2.7. Ethical Considerations

Ethical approval for this study was obtained from The Seventh Clinical Medical College of Guangzhou University of Chinese Medicine, Shenzhen, China (ethical review no. KY-2023-125-01), based on the principles of the Declaration of Helsinki. All eligible nurses were informed of the study and its ethical principles (e.g., voluntary participation, withdrawal, anonymity, and confidentiality). Prior to data collection, informed consent was obtained from all participants, who were provided with both oral and written explanations of the purpose and procedures of the study. Participants were fully informed that their participation was voluntary and that they could withdraw from the study at any length.

To ensure confidentiality and anonymity, no identifying information, such as names, was collected. At the same time, each questionnaire was numbered, and participants were provided with a corresponding number on a debriefing sheet. This allowed for the maintenance of confidentiality throughout the study. Additionally, participants were informed that they could choose to withdraw their data prior to data analysis by contacting the researcher. Furthermore, all collected data were stored securely. Electronic data were stored on a password-protected computer to ensure the privacy and security of participants' information.

## 3. Results

### 3.1. Social–Demographic Characteristics of Participants

The social–demographic characteristics of participants and the difference in compassion fatigue are shown in Table 1. Most of the nurses were females (1253, 95.285%) and aged 26 to 35 years (860, 65.399%). Most of them are married (944, 71.787%), worked for 10 to 20 years (507, 38.555%), and had a bachelor's degree (1166, 88.669%). Organizational affiliations of nurses were mainly human agency (634, 48.213%). 677 nurses had junior titles (51.483%), and 1036 nurses were general nurses (78.783%). 523 (39.772%) nurses had no night shift.

### 3.2. Common Method Bias

In this study, the influence of common method bias was minimized via anonymous filling and concealing variable names. Harman's single-factor test showed that the variance explained by the first eigenvalue was 38.949%, which was < 40%, indicating that the common method bias of this study was acceptable [68].

### 3.3. Correlation Study

Table 2 provides the means, standard deviations, and correlations among the variables. The results showed that mindful self-care was positively correlated with resilience (*r* = 0.409, *p* < 0.01) and professional identity (*r* = 0.730, *p* < 0.01) and negatively correlated with compassion fatigue (*r* = −0.432, *p* < 0.01). Resilience was positively correlated with professional identity (*r* = 0.556, *p* < 0.01) and negatively correlated with compassion fatigue (*r* = −0.559, *p* < 0.01). Professional identity was negatively correlated with compassion fatigue (*r* = −0.501, *p* < 0.01).

### 3.4. Chain Mediation Model Analysis

A serial mediation analysis was conducted to test the effect of resilience and professional identity as multiple sequential mediators in the indirect relationship between mindful self-care and compassion fatigue in nurses via Model 6 in the SPSS macro. Mindful self-care and compassion fatigue were entered as the independent (X) and dependent (Y) variables, respectively; resilience (M1) and professional identity (M2) were added as mediators; gender, age, marital status, working years, educational attainment, organizational affiliation, professional title, position title, and night shift were included as covariates.

**Figure 3:**
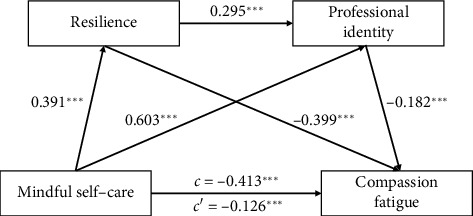
Standardized regression coefficients in the chain mediation model. *c*, total effect; *c*′, direct effect; ⁣^∗∗∗^*p* < 0.001. Gender, age, marital status, working years, educational attainment, organizational affiliation, professional title, position title, and night shift were treated as covariates in the chain mediation model. Path coefficients are standardized.

As shown in Table 3 and Figure 3, mindful self-care had a significant negative impact on a nurse's compassion fatigue, which supported Hypothesis 1. The total effect of mindful self-care on compassion fatigue (*β* = −0.413, *p* < 0.001) decreased when the mediators were included in the model (*β* = −0.126, *p* < 0.001), suggesting that the effect of mindful self-care on compassion fatigue was partially mediated by resilience and professional identity.

The bootstrap estimation procedure (*n* = 5000) indicated that the indirect effects for this model were statistically significant (indirect effect = −0.409, SE = 0.050, and 95% CI = [−0.506, −0.309], contributing 69.322% of the total effect; Table 3). The indirect effects were generated through three paths: Path 1 consists of mindful self-care ⟶ resilience ⟶ compassion fatigue (indirect effect = −0.223 and 95% CI = [−0.286, −0.169]), confirming the significant mediating role of resilience and supporting Hypothesis 2. Path 2 consists of mindful self-care ⟶ professional identity ⟶ compassion fatigue (indirect effect = −0.157 and 95% CI [−0.248, −0.062]), confirming the significant mediating role of professional and supporting Hypothesis 3. Path 3 consists of mindful self-care ⟶ resilience ⟶ professional identity ⟶ compassion fatigue (indirect effect = −0.030 and 95% CI [−0.047, −0.013]), confirming significant serial mediation of resilience and professional identity and supporting Hypothesis 4.

## 4. Discussion

This study investigated the impact of mindful self-care on compassion fatigue among a sample of Chinese nurses, as well as the mediating role played by resilience and professional identity. The results revealed that mindful self-care not only directly but also indirectly through resilience and professional identity separately or jointly influenced compassion fatigue. By exploring the underlying mechanisms of the impact of positive self-care on compassion fatigue, the present study may provide a new insight into current perceptions of nurses' compassion fatigue.

The average compassion fatigue score observed in this study (45.370 ± 22.877) was significantly lower than the results obtained by Çiçek Korkmaz and Gökoğlan [69] and Sahin et al. [70], likely because both studies were conducted during the COVID-19 pandemic, a period characterized by heightened emotional exhaustion and work-related stress among nurses [71]. Conversely, our findings align with another study conducted in China [72]. The relatively low compassion fatigue scores observed in this study may also reflect the collectivist cultural context in China, where nurses tend to benefit from enhanced teamwork and social support [73, 74].

In the present study, we found a strong negative association between mindful self-care and compassion fatigue, which was consistent with previous studies [24, 75], further highlighting the crucial role of mindful self-care in preventing and alleviating compassion fatigue. In particular, in the present study, we validated this association in a large number of Chinese nurses, extending the generalizability, specificity, and local validity of this association. These findings align with Cook-Cottone's framework, highlighting mindful self-care's role in fostering emotional regulation and reducing compassion fatigue [19, 24]. Prior research has found that mindful self-care behaviors had a direct effect on professional quality of life [15], which includes compassion fatigue as one dimension. From a humanistic nursing perspective, nurses were often faced with significant emotional labor demands, as they need to develop deep emotional connections with patients and their families while providing patient-centered care [76]. It seemed to create a paradox that sustained emotional labor was the foundation of compassionate care, but it also exposed nurses to a higher risk of compassion fatigue, especially when emotional resources are depleted without adequate recovery mechanisms. Mindful self-care served as a protective mechanism against these challenges by promoting emotional regulation, cultivating self-compassion, and increasing awareness of one's demands [19]. What is more, according to the broaden-and-build theory proposed by Fredrickson [77], nurses experience compassion fatigue or compassion satisfaction depending on the proportion of positive and negative emotions felt while helping others [78]. Research suggests that mindful self-care is positively associated with the cultivation of positive emotions, such as gratitude and compassion satisfaction [15], and negatively correlated with negative emotional states, such as stress, anxiety, and depression [79]. Therefore, mindful self-care appears to serve as a mechanism for regulating the balance between positive and negative emotions in high-stress caregiving roles. Although mindful self-care is widely recognized as beneficial in reducing compassion fatigue, its effectiveness may depend on environmental factors such as workplace culture or workplace organizational factors. Future research could explore how organizational and cultural factors influence the relationship between mindful self-care and compassion fatigue.

In addition to the observed direct effects, the present study identified a partial mediating role of resilience and professional identity between mindful self-care and compassion fatigue. The mediating effect value accounted for 69.322% of the total effect. Based on the mediating role of resilience in the relationship between mindful self-care and compassion fatigue, we found that resilience has a protective effect on nurses' compassion fatigue. Within the framework of PsyCap theory, resilience is conceptualized as a core component of positive psychological resources that are both measurable and malleable [80]. PsyCap theory emphasizes that resilience can be developed through targeted interventions, such as mindful self-care, which can enhance emotional regulation and equip individuals to recover from adversity [32, 81]. From the perspective of JD-R theory, mindful self-care practices can be understood as proactive, resource-building behaviors that strengthen resilience. JD-R theory proposes that adequate psychological resources, such as resilience, can buffer the negative effects of high job demands, such as burnout [27]. Research shows that nurses with higher levels of resilience were less likely to experience compassion fatigue [31]. It may be that resilient nurses are better equipped to adapt to workplace challenges, such as high emotional demands and compassion fatigue triggers, by reframing adversity as an opportunity for growth [30]. By increasing resilience, mindful self-care enables nurses to better manage emotional responses and recover more quickly from occupational stress to better adapt to workplace challenges, which consequently reduces the risk of developing compassion fatigue. Previous research has found that improving self-care and resilience were effective strategies to prevent compassion fatigue [82–84]. Overall, these findings emphasize the importance of resilience as a key psychological resource for reducing compassion fatigue and promoting nurses' well-being. By promoting mindful self-care practices as a proactive approach to resource building, healthcare organizations can enhance nurses' resilience and emotional management skills, which in turn reduces their risk of compassion fatigue.

We also found that professional identity mediated the relationship between mindful self-care and compassion fatigue. Specifically, mindful self-care was positively correlated to professional identity, and professional identity was negatively correlated to compassion fatigue. These findings were consistent with Social Identity Theory. According to the Social Identity Theory, professional identity fosters a sense of belonging, meaning, and purpose, which can buffer the emotional toll of compassion-demanding roles. For nurses working in environments that require compassion, a strong professional identity provides a framework for deriving positive meaning from their work, which can mitigate the emotional toll associated with compassion fatigue [85]. Professional identity helps nurses reframe professional challenges as meaningful and manageable experiences rather than overwhelming stressors. Research has shown that people with higher levels of professional identity are more likely to view their work positively and find fulfillment even in high-pressure situations [86]. For example, it has been shown that professional identity acts as a partial mediator between moral distress and compassion fatigue, reducing the negative effects of distress by providing a stronger sense of purpose and alignment with occupational values [87]. This emphasizes the importance of professional identity as a psychological resource that can help nurses maintain their psychological well-being in the face of chronic emotional demands. Taken together, our findings highlight the mediating role of professional identity between mindful self-care and compassion fatigue, emphasizing its importance in reducing psychological stress in compassionate demand occupations.

Furthermore, this study demonstrated that resilience and professional identity played a chain mediating role between mindful self-care and compassion fatigue. This finding suggests that mindful self-care as an effective measure to build psychological resources promotes resilience, subsequently enhances professional identity, and ultimately reduces compassion fatigue in nurses. Although these chain mediating effects had not been examined in previous studies, Cui et al. [88] observed that resilience and professional identity were negatively correlated with fatigue. Moreover, several studies have found that mindful self-care positively affects resilience [32, 81, 89] and protects individuals from compassion fatigue [23, 24]. The sequential relationship between resilience and professional identity emphasizes the dynamic and cumulative nature of these psychological resources. Mindful self-care builds resilience by fostering emotional awareness and self-regulation, which in turn enhances nurses' ability to align their values and actions with their professional roles. This alignment not only reduces the risk of compassion fatigue but also contributes to mental health and professional satisfaction. Importantly, the interlocking mediating effects of resilience and professional identity may be particularly pronounced in high-stress healthcare environments [90], where both resources are critical to maintaining mental health and providing compassionate care. There is evidence that resilience helps to maintain optimistic thinking and promote self-reflection and self-worth in the nursing profession and thus improve the professional identity of nurses [91]. Thirdly, a higher level of professional identity can protect nurses from compassion fatigue. The reason may be that nurses with higher levels of professional identity are more likely to give positive meaning to the helping situation, reduce the generation of negative emotions, and then protect nurses from compassion fatigue [87]. Future research should examine how interventions targeting mindful self-care can systematically enhance both resilience and professional identity. Additionally, longitudinal studies are needed to explore how these relationships evolve over time and across different healthcare settings and cultural contexts. This integrated perspective offers valuable insights for developing comprehensive strategies to address compassion fatigue and promote well-being among healthcare professionals.

## 5. Limitations

A few limitations were identified in our study and are unavoidable. First of all, the cross-sectional design used in the present study made the causality cannot be inferred and make it could not discuss the long-term effects between variables. Thus, the current findings need to be interpreted with caution, and further research is needed to examine the feasibility of using the findings in care management. Future research should conduct longitudinal studies to identify how the relationships between these variables change over time. Secondly, the method of convenience sampling used in this study may limit the generalization of the results, which makes this study less representative, although the samples are relatively easy to collect. Nurses from several tertiary hospitals from three different provinces in China were selected for this study through convenience sampling only, and future studies need to expand the survey area (e.g., other regions of China or other countries) to improve the generalizability of the results. Thirdly, male nurses account for too few in our sample of study participants, making the results less representative. In the future, intervention studies can be adopted in further studies to verify how much mitigation effect mindful self-care has on nurses' compassion fatigue. Last but not least, there may be the possibility of other independent variables being missed; for example, we did not collect information related to hospital characteristics such as different provinces, number of beds, annual patient volume, and affiliation to universities in this study, but these characteristics may indeed affect the measurements of the study variables to some extent. Exploring the effects of these hospital characteristics on the study variables could be a key direction for future research.

## 6. Conclusion

The results support our hypothesis that in the nurse group, mindful self-care is negatively correlated with compassion fatigue, and resilience and professional identity have a chain mediating effect between these two variables. At the same time, our results showed that mindful self-care, resilience, and professional identity could protect nurses from compassion fatigue. This study offers valuable insights for hospital administrators and policymakers to reduce nurses' compassion fatigue and improve workplace well-being. This study can provide a basis for nurse managers to improve the quality of nursing services and reduce the compassion fatigue of clinical nurses. Further research should carry out clinical trials to test whether the training courses based on the improvement of mindful self-care ability are effective in improving nurses' resilience and professional identity and reducing compassion fatigue, and improving the specific course content and course design is also necessary.

## 7. Implications for Nursing Management

The significant contribution of our study is the finding that mindful self-care could directly and indirectly affect nurses' compassion fatigue via resilience and professional identity, suggesting the importance of positive mental status in the prevention of compassion fatigue during the work of nurses. Recognizing these not only highlights insights into the valuable protective role of positive mental status in the prevention of compassion fatigue but also serves as a foundation for developing tailored interventions and support systems. Therefore, to protect nurses from compassion fatigue, it is necessary to improve nurses' ability of mindful self-care, enhance nurses' resilience, and enhance nurses' professional identity.

On the one hand, considering the importance of mindful self-care, nurse managers should take measures to help nurses improve the frequency and ability of mindful self-care. For example, a brief psychosocial intervention based on mindful self-care can be carried out to improve nurses' self-awareness, reflection, and self-care skills [89, 92]. At the same time, nurse leaders can consider establishing a special activity room in the hospital for meditation or carrying out mindful self-care-related activities. In addition, nurse leaders can consider to establish a special activity room in the hospital for meditation or carrying out mindful self-care-related activities to provide a favorable environment for nurses to practice mindful self-care more frequently.

On the other hand, the mediating role of resilience and professional identity highlights the importance of a positive mental state as an important psychological resource to protect nurses from compassion fatigue. In view of this, nurse leaders should take the cultivation of nurses' positive psychological resources as an important part of nurse training. In the past, nurse training seemed to focus only on nurses' operational skills and judgment ability. In fact, this kind of ability, such as the ability to accumulate psychological resources or the ability to mobilize psychological resources to protect themselves from being in a bad psychological state in a stressful working environment, is equally important for nurses.

All in all, hospital leaders should actively pay attention to the psychological state of nurses and take measures to improve their ability of mindful self-care, which will help to prevent compassion fatigue of nurses and improve the quality of nursing services.

## Figures and Tables

**Table 1:** Social–demographic characteristics of participants (*N* = 1315).

Variables	Category	*n*	Percentage (%)
Gender	Male	62	4.715
	Female	1253	95.285

Age	< 25 years old	127	9.658
	26∼35 years old	860	65.399
	> 36 years old	328	24.943

Marital status	Married	944	71.787
	Unmarried	350	26.616
	Divorced	19	1.445
	Widowed	2	0.152

Working years	< 2 years	95	7.224
	2∼5 years	195	14.829
	6∼10 years	428	32.548
	10∼20 years	507	38.555
	≥ 20 years	90	6.844

Educational attainment	Junior college's degree	139	10.570
	Bachelor's degree	1166	88.669
	Master's degree	8	0.608
	Doctoral degree	2	0.152

Organizational affiliation	Personnel on payroll	448	34.068
	Human agency	634	48.213
	Contract labor	233	17.719

Professional title	Junior title	677	51.483
	Intermediate title	586	44.563
	Deputy senior title	46	3.498
	Senior title	6	0.456

Position title	General nurse	1036	78.783
	Responsible group leader	189	14.372
	Head nurse	86	6.540
	Director/deputy director of nursing	4	0.304

Night shift	None	523	39.772
	1∼3 times/month	103	7.833
	4∼6 times/month	251	19.087
	≥ 7 times/month	438	33.308

Abbreviation: *n*, number.

**Table 2:** Correlations among mindful self-care, resilience, professional identity, and compassion fatigue.

Variables	*M*	SD	1	2	3	4
1. Mindful self-care	75.325	16.025	1			
2. Resilience	62.073	16.983	0.409⁣^∗∗^	1		
3. Professional identity	101.087	22.192	0.730⁣^∗∗^	0.556⁣^∗∗^	1	
4. Compassion fatigue	45.370	22.877	−0.432⁣^∗∗^	−0.559⁣^∗∗^	−0.501⁣^∗∗^	1

Abbreviations: *M*, mean; SD, standard deviation.

⁣^∗∗^*p* < 0.01.

**Table 3:** Total, direct, and indirect effects of the mediation model.

Effect	Product of coefficients		Bootstrapping 95% CI	
	Point estimate	Boot SE	Lower	Upper
Total effect of MS on CF	−0.590⁣^∗∗∗^	0.036	−0.660	−0.520
Direct effect of MS on CF	−0.180⁣^∗∗∗^	0.046	−0.270	−0.091
Total indirect effect of MS on CF	−0.409⁣^∗∗∗^	0.050	−0.506	−0.309
Indirect 1: MS ⟶ R ⟶ CF	−0.223⁣^∗∗∗^	0.030	−0.286	−0.169
Indirect 2: MS ⟶ PI ⟶ CF	−0.157⁣^∗∗∗^	0.048	−0.248	−0.062
Indirect 3: MS ⟶ R ⟶ PI ⟶ CF	−0.030⁣^∗∗∗^	0.009	−0.047	−0.013

*Note:* Gender, age, marital status, working years, educational attainment, organizational affiliation, professional title, position title, and night shift were treated as covariates in the chain mediation model. All paths are expressed as unstandardized regression coefficients.

Abbreviations: CI, confidence interval; SE, standard error.

⁣^∗∗∗^*p* < 0.001.

## Data Availability

The data that support the findings of this study are available from the corresponding author upon reasonable request.

## Supporting Information

Supporting 1: Table S1. STROBE statement: Checklist of items that should be included in reports of cross-sectional studies.

## References

[1] Lown B. A. (2015). Compassion Is a Necessity and an Individual and Collective Responsibility Comment on ‘Why and How Is Compassion Necessary to Provide Good Quality Healthcare?’. *International Journal of Health Policy and Management*.

[2] Gallagher A., Timmins F. (2022). Admission to Undergraduate Nurse Education Programmes: Who Should Be Selected?. *Nursing Ethics*.

[3] Schantz M. L. (2007). Compassion: A Concept Analysis. *Nursing Forum*.

[4] International Council of Nursing (2012). The ICN Code of Ethics for Nurses. https://www.icn.ch/who-we-are/code-of-ethics-for-nurses/.

[5] Strauss C., Lever Taylor B., Gu J. (2016). What Is Compassion and How Can We Measure It? A Review of Definitions and Measures. *Clinical Psychology Review*.

[6] Sacco T. L., Copel L. C. (2018). Compassion Satisfaction: A Concept Analysis in Nursing. *Nursing Forum*.

[7] Bilgiç Ş. (2022). Does the Compassion Level of Nursing Students Affect Their Ethical Sensitivity?. *Nurse Education Today*.

[8] Duarte J., Pinto-Gouveia J. (2016). Effectiveness of a Mindfulness-Based Intervention on Oncology Nurses’ Burnout and Compassion Fatigue Symptoms: A Non-Randomized Study. *International Journal of Nursing Studies*.

[9] Figley C. R. (2002). Compassion Fatigue: Psychotherapists’ Chronic Lack of Self Care. *Journal of Clinical Psychology*.

[10] Peters E. (2018). Compassion Fatigue in Nursing: A Concept Analysis. *Nursing Forum*.

[11] Milutinović D., Marcinowicz L., Jovanović N. B., Dragnić N. (2023). Impact of Compassion Satisfaction and Compassion Fatigue on Satisfaction With Life in Serbian and Polish Nurses: A Cross-Sectional Study. *International Nursing Review*.

[12] Cao X., Chen L. (2021). Relationships between Resilience, Empathy, Compassion Fatigue, Work Engagement and Turnover Intention in Haemodialysis Nurses: A Cross-Sectional Study. *Journal of Nursing Management*.

[13] Xia W., Defang W., Xiaoli G. (2022). Compassion Satisfaction and Compassion Fatigue in Frontline Nurses During the COVID-19 Pandemic in Wuhan, China. *Journal of Nursing Management*.

[14] World Health Organization State of the Worlds’ Nursing 2020: Executive Summary. https://apps.who.int/iris/handle/10665/331673.

[15] Halady E., Cook-Cottone C. (2023). Mindful Self-Care, Coping, and Meaning in Life: An Examination of the Professional Quality of Life and Well-Being Among Individuals Who Support and Provide Services to Refugees. *Psychological Trauma: Theory, Research, Practice, and Policy*.

[16] Hotchkiss J. T., Cook-Cottone C. (2023). The Mindful Helping and Self-Care Model: Mindful Self-Care and Quality of Life Among a Racially Balanced Sample of Helping Professionals. *International Journal of Yoga Therapy*.

[17] Mcross M. C. (1992). Coping With Compassion Fatigue. *Nursing*.

[18] Hotchkiss J. T., Cook-Cottone C. P. (2019). Validation of the Mindful Self-Care Scale (MSCS) and Development of the Brief-MSCS Among Hospice and Healthcare Professionals: A Confirmatory Factor Analysis Approach to Validation. *Palliative & Supportive Care*.

[19] Cook-Cottone C. P. (2015). Incorporating Positive Body Image into the Treatment of Eating Disorders: A Model for Attunement and Mindful Self-Care. *Body Image*.

[20] Alkema K., Linton J. M., Davies R. (2008). A Study of the Relationship between Self-Care, Compassion Satisfaction, Compassion Fatigue, and Burnout Among Hospice Professionals. *Journal of Social Work in End-of-Life and Palliative Care*.

[21] Gantt L. T., Haberstroh A. L. (2023). Nurses’ Self-Care Strategies: A Mapping Review. *Worldviews on Evidence-Based Nursing*.

[22] Stephen J. S. (2024). Mindfulness, Self-Care, and Stress Management. *Academic Success in Online Programs: A Resource for College Students*.

[23] Hotchkiss J. T. (2018). Mindful Self-Care and Secondary Traumatic Stress Mediate a Relationship Between Compassion Satisfaction and Burnout Risk Among Hospice Care Professionals. *American Journal of Hospice and Palliative Medicine*.

[24] Hotchkiss J. T., Lesher R. (2018). Factors Predicting Burnout Among Chaplains: Compassion Satisfaction, Organizational Factors, and the Mediators of Mindful Self-Care and Secondary Traumatic Stress. *Journal of Pastoral Care and Counseling*.

[25] Connor K. M., Davidson J. R. (2003). Development of a New Resilience Scale: The Connor-Davidson Resilience Scale (CD-RISC). *Depression and Anxiety*.

[26] Shatté A., Perlman A., Smith B., Lynch W. D. (2017). The Positive Effect of Resilience on Stress and Business Outcomes in Difficult Work Environments. *Journal of Occupational and Environmental Medicine*.

[27] Demerouti E., Bakker A. B., Nachreiner F., Schaufeli W. B. (2001). The Job Demands-Resources Model of Burnout. *Journal of Applied Psychology*.

[28] Kuhlmann R., Süß S. (2024). The Dynamic Interplay of Job Characteristics and Psychological Capital With Employee Health: A Longitudinal Analysis of Reciprocal Effects. *Journal of Occupational Health Psychology*.

[29] Ueno Y., Amemiya R. (2024). Mediating Effects of Resilience Between Mindfulness, Self-Compassion, and Psychological Distress in a Longitudinal Study. *Journal of Rational-Emotive and Cognitive-Behavior Therapy*.

[30] Foster K., Roche M., Delgado C., Cuzzillo C., Giandinoto J. A., Furness T. (2019). Resilience and Mental Health Nursing: An Integrative Review of International Literature. *International Journal of Mental Health Nursing*.

[31] Marshman C., Hansen A., Munro I. (2022). Compassion Fatigue in Mental Health Nurses: A Systematic Review. *Journal of Psychiatric and Mental Health Nursing*.

[32] Garcia A. C. M., Ferreira A. C. G., Silva L. S. R., da Conceição V. M., Nogueira D. A., Mills J. (2022). Mindful Self-Care, Self-Compassion, and Resilience Among Palliative Care Providers During the COVID-19 Pandemic. *Journal of Pain and Symptom Management*.

[33] Li M., Wei J., Yang S. (2024). Relationships Among Perceived Social Support, Mindful Self-Care, and Resilience Among a Sample of Nurses in Three Provinces in China: A Cross-Sectional Study. *Frontiers in Public Health*.

[34] Leong Y. M., Crossman J. (2015). New Nurse Transition: Success Through Aligning Multiple Identities. *Journal of Health, Organisation and Management*.

[35] Wrzesniewski A., Mccauley C., Rozin P., Schwartz B. (1997). Jobs, Careers, and Callings: People’s Relations to Their Work. *Journal of Research in Personality*.

[36] Shuai T., Xuan Y., Jiménez-Herrera M. F., Yi L., Tian X. (2024). Moral Distress and Compassion Fatigue Among Nursing Interns: A Cross-Sectional Study on the Mediating Roles of Moral Resilience and Professional Identity. *BMC Nursing*.

[37] Saribudaka T. P., Ustun B. (2024). Compassion Fatigue Resiliency Program Effects on Oncology-Hematology Nurses’ Professional Quality of Life, Stress Levels, and Patients’ Care Satisfaction: Nurse, Nurse Manager, and Patient Perspectives, a Mixed Methods. *Seminars in Oncology Nursing*.

[38] Geoffrion S., Lamothe J., Giguère C., Collin-Vézina D. (2023). The Effects of Adherence to Professional Identity, Workplace Aggression, and Felt Accountability on Child Protection Workers’ Professional Quality of Life. *Child Abuse & Neglect*.

[39] Bhattarai M., Clements P. T., Downing N. R. (2024). Mindfulness-Based Self-Care for Forensic Nurses: A Professional Lifestyle Approach. *Journal of Forensic Nursing*.

[40] Burgess D. J., Beach M. C., Saha S. (2017). Mindfulness Practice: A Promising Approach to Reducing the Effects of Clinician Implicit Bias on Patients. *Patient Education and Counseling*.

[41] Scholes J. (2008). Coping with the Professional Identity Crisis: Is Building Resilience the Answer?. *International Journal of Nursing Studies*.

[42] Stevens M. J. (1992). Prescott Lecky: Pioneer in Consistency Theory and Cognitive Therapy. *Journal of Clinical Psychology*.

[43] Atay N., Sahin G., Buzlu S. (2021). The Relationship Between Psychological Resilience and Professional Quality of Life in Nurses. *Journal of Psychosocial Nursing and Mental Health Services*.

[44] Li Y. R., Liu J. Y., Fang Y., Shen X., Li S. W. (2024). Novice Nurses’ Transition Shock and Professional Identity: The Chain Mediating Roles of Self-Efficacy and Resilience. *Journal of Clinical Nursing*.

[45] Yi R., Zhou Z., Ma W., Yang C., Wang F., Wu J. (2024). Mediating Role of Psychological Resilience in the Relationship Between Self-Efficacy and Professional Identity Among Nurses. *Biotechnology & Genetic Engineering Reviews*.

[46] Wang X., Cheng Z. (2020). Cross-Sectional Studies: Strengths, Weaknesses, and Recommendations. *Chest*.

[47] Rodríguez Del Águila M., González-Ramírez A. (2014). Sample Size Calculation. *Allergologia et Immunopathologia*.

[48] Faul F., Erdfelder E., Buchner A., Lang A. G. (2009). Statistical Power Analyses Using G∗Power 3.1: Tests for Correlation and Regression Analyses. *Behavior Research Methods*.

[49] Perneger T. V., Courvoisier D. S., Hudelson P. M., Gayet-Ageron A. (2015). Sample Size for Pre-Tests of Questionnaires. *Quality of Life Research*.

[50] Park M. S., Kang K. J., Jang S. J., Lee J. Y., Chang S. J. (2018). Evaluating Test-Retest Reliability in Patient-Reported Outcome Measures for Older People: A Systematic Review. *International Journal of Nursing Studies*.

[51] Hunsaker S., Chen H. C., Maughan D., Heaston S. (2015). Factors That Influence the Development of Compassion Fatigue, Burnout, and Compassion Satisfaction in Emergency Department Nurses. *Journal of Nursing Scholarship*.

[52] Storm J., Chen H. C. (2021). The Relationships Among Alarm Fatigue, Compassion Fatigue, Burnout and Compassion Satisfaction in Critical Care and Step-Down Nurses. *Journal of Clinical Nursing*.

[53] Xie W., Wang J., Zhang Y. (2021). The Levels, Prevalence and Related Factors of Compassion Fatigue Among Oncology Nurses: A Systematic Review and Meta-Analysis. *Journal of Clinical Nursing*.

[54] Cook-Cottone C. P., Guyker W. M. (2018). The Development and Validation of the Mindful Self-Care Scale (MSCS): An Assessment of Practices that Support Positive Embodiment. *Mindfulness*.

[55] Yang Z., Chen F., Liu S., Dai M., Zhang H. (2021). Psychometric Properties of the Chinese Version of the Brief-Mindful Self-Care Scale: A Translation and Validation Study. *Frontiers in Psychology*.

[56] Zhang Y., Wang T., Jin S., Zhang H., Chen L., Du S. (2023). Resilience Mediates the Association Between Alexithymia and Stress in Chinese Medical Students During the COVID-19 Pandemic. *General Psychiatry*.

[57] Ong H. L., Vaingankar J. A., Abdin E. (2018). Resilience and Burden in Caregivers of Older Adults: Moderating and Mediating Effects of Perceived Social Support. *BMC Psychiatry*.

[58] Koprowski K., Meyer D., Stanfill T., Tivis L. J. (2021). Cultivating Joy: Improving Nurse Resilience Through Use of a Practice Playbook. *Applied Nursing Research*.

[59] Yu X. N., Lau J. T., Mak W. W., Zhang J., Lui W. W., Zhang J. (2011). Factor Structure and Psychometric Properties of the Connor-Davidson Resilience Scale Among Chinese Adolescents. *Comprehensive Psychiatry*.

[60] Liu L., Zhang Y., Liu X. (2009). Study on the Correlation Between Nurses’ Professional Identity Level and Their Job Stress and Job Burnout. *The Journal of Nursing Administration*.

[61] Adams R. E., Figley C. R., Boscarino J. A. (2008). The Compassion Fatigue Scale: Its Use With Social Workers Following Urban Disaster. *Research on Social Work Practice*.

[62] Lou B. (2012). *The Structure and Mechanism of Compassion Fatigue: An Examination Based on Different Helping Groups*.

[63] Gilvari T., Babamohamadi H., Paknazar F. (2022). Perceived Professional Identity and Related Factors in Iranian Nursing Students: A Cross-Sectional Study. *BMC Nursing*.

[64] Podsakoff P. M., MacKenzie S. B., Lee J. Y., Podsakoff N. P. (2003). Common Method Biases in Behavioral Research: A Critical Review of the Literature and Recommended Remedies. *Journal of Applied Psychology*.

[65] Podsakoff P. M., MacKenzie S. B., Podsakoff N. P. (2012). Sources of Method Bias in Social Science Research and Recommendations on How to Control it. *Annual Review of Psychology*.

[66] Igartua J. J., Hayes A. F. (2021). Mediation, Moderation, and Conditional Process Analysis: Concepts, Computations, and Some Common Confusions. *Spanish Journal of Psychology*.

[67] Preacher K. J., Hayes A. F. (2008). Asymptotic and Resampling Strategies for Assessing and Comparing Indirect Effects in Multiple Mediator Models. *Behavior Research Methods*.

[68] Tang D., Wen Z. (2020). Statistical Approaches for Testing Common Method Bias: Problems and Suggestions. *Journal of Psychological Science*.

[69] Çiçek Korkmaz A., Gökoğlan E. (2023). Do Nurses’ Personality Traits Affect the Level of Compassion Fatigue?. *International Nursing Review*.

[70] Sahin S., Arioz Duzgun A., Unsal A., Inan Kirmizigul E., Ozdemir A. (2023). Assessment of Compassion Fatigue and Empathy Levels in Nurses during the COVID-19 Outbreak: Turkey’s Case. *Journal of Religion and Health*.

[71] Hawsawi S. (2022). Alleviating Psychological Symptoms in Nurses During Disease Outbreaks: An Integrative Review. *International Nursing Review*.

[72] Chuanru Z., Xia H., Cong W., Ting Y., Yan J. (2022). The Current Status and Influencing Factors of Caring Behavior Among ICU Nurses in 24 Tertiary Hospitals in Sichuan Province. *Chinese Journal of Nursing*.

[73] Kuah-Pearce K. E., Kleinman A., Harrison E. (2014). Social Suffering and the Culture of Compassion in a Morally Divided China. *Anthropology & Medicine*.

[74] Liu X., Li J., Wang S. (2024). The Impact of Grit on Nurses’ Job Performance: Evaluating Chained Mediation Through Perceived Social Support and Self-Esteem. *Journal of Nursing Management*.

[75] McMakin D., Ballin A., Fullerton D. (2022). Secondary Trauma, Burnout, and Teacher Self-Care During COVID19: A Mixed-Methods Case Study. *Psychology in the Schools*.

[76] Wang Y. L., Yang Z. W., Tang Y. Z., Li H. L., Zhou L. S. (2022). A Qualitative Exploration of “Empathic Labor” in Chinese Hospice Nurses. *BMC Palliative Care*.

[77] Fredrickson B. L. (1998). What Good Are Positive Emotions?. *Review of General Psychology*.

[78] Diener E. (2000). Subjective Well-Being. The Science of Happiness and a Proposal for a National Index. *American Psychologist*.

[79] Xavier S., Branquinho M., Pires R., Moreira H., Coelho M., Araújo-Pedrosa A. (2023). Dysfunctional Attitudes Toward Motherhood and Depressive Symptoms in Portuguese Pregnant Women During COVID-19 Pandemic: The Mediating Roles of Self-Compassion and Mindful Self-Care. *Mindfulness (N Y)*.

[80] Luthans F., Youssef-Morgan C. M., Avolio B. J. (2007). Psychological Capital: Developing the Human Competitive Edge. *Journal of Asian Economics*.

[81] Zeb H., Arif I., Younas A. (2022). Mindful Self-Care Practice of Nurses in Acute Care: A Multisite Cross-Sectional Survey. *Western Journal of Nursing Research*.

[82] Li J.-N., Jiang X.-M., Zheng Q.-X. (2023). Mediating Effect of Resilience Between Social Support and Compassion Fatigue Among Intern Nursing and Midwifery Students During COVID-19: A Cross-Sectional Study. *BMC Nursing*.

[83] Roussel L. A. (2022). On-the-Go Strategies to Enhance Resilience and Self-Care: Using Technology to Create Healthy Work Cultures. *Nursing Clinics of North America*.

[84] Shuai T., Yi L., Xuan Y., Jiménez-Herrera M. F., Tian X. (2024). Mediating Role of Moral Resilience and Professional Identity in the Impact of Nursing Interns’ Moral Distress on Compassion Fatigue: A Cross-Sectional Study.

[85] Al-Sabiry M., Barnhoorn P., Slootweg I., van Mook W., Numans M. (2024). Which ‘End’ Do You Have in Mind? Clinical Supervisors’ Perceptions of Professional Identity Formation Outcomes in GP Residency. *Medical Teacher*.

[86] Byrne V. (2011). Emotion and Professional Identities: A Comparative Study of Professionals in Further Education and Learning Disability Support Services.

[87] Geoffrion S., Morselli C., Guay S. (2016). Rethinking Compassion Fatigue through the Lens of Professional Identity: The Case of Child-Protection Workers. *Trauma, Violence, & Abuse*.

[88] Cui Q., Liu L., Hao Z. (2022). Research on the Influencing Factors of Fatigue and Professional Identity Among CDC Workers in China: An Online Cross-Sectional Study. *BMJ Open*.

[89] Rees C. S., Craigie M. A., Slatyer S. (2020). Pilot Study of the Effectiveness of a Mindful Self-Care and Resiliency Program for Rural Doctors in Australia. *Australian Journal of Rural Health*.

[90] Cooper A. L., Brown J. A., Rees C. S., Leslie G. D. (2020). Nurse Resilience: A Concept Analysis. *International Journal of Mental Health Nursing*.

[91] Shi Y., Zhou J. X., Shi J. L., Pan J. F., Dai J. Y., Gao Q. (2023). Association between Proactive Personality and Professional Identity of Nursing Undergraduates: The Mediating Role of Resilience and Irrational Belief. *Nurse Education in Practice*.

[92] Rees C., Craigie M., Slatyer S. (2018). Mindful Self-Care and Resiliency (MSCR): Protocol for a Pilot Trial of a Brief Mindfulness Intervention to Promote Occupational Resilience in Rural General Practitioners. *BMJ Open*.

